# Supplementary material for: Prognosis of Stage I Endometrial Cancer According to the FIGO 2023 Classification Taking into Account Molecular Changes
Source: Cancers (Basel). 2024 Jan 17;16(2):390. doi: 10.3390/cancers16020390 (PMC10813919; doi:10.3390/cancers16020390)
Supplement: Supplementary file 1 [file cancers-16-00390-s001.zip › cancers-2750054-supplementary.pdf]

Table S1. Primer nucleotide sequences used to amplify *POLE* exons 9-14.

Primer Name	Primer Sequence
9_F	gttcagggaggcctaatggg
9_R	actaacagtggggcagatgc
10_F	gctttgcagcctgacttg
10_R	ccacatgtccgttcttccca
11_1F	gcttctgaactttgggagagga
11_R	aacgccctccctcaaatg
12_F	ggaggaatggagaaaggggc
12_R	gaggccttcagatctcgctc
13_F	tctgtgtggtgcccagtttt
13_R	ctccgtggccatctggatg
14_F	ctgtgccggtctccttactg
14_R	ctccattcagctccagtgca
